# (Cobaltoceniumyl­amido)­pyridinium hexa­fluorido­phosphate

**DOI:** 10.1107/S2414314621004600

**Published:** 2021-07-05

**Authors:** Daniel Menia, Thomas Höfer, Klaus Wurst, Benno Bildstein

**Affiliations:** a University of Innsbruck, Faculty of Chemistry and Pharmacy, Innrain 80-82, 6020 Innsbruck, Austria; Howard University, USA

**Keywords:** cobalt, cobaltocenium, ylide, microwave-assisted synthesis, crystal structure

## Abstract

The title compound, [Co(C_5_H_5_)(C_10_H_9_N_2_)]PF_6_, was synthesized from deproton­ated 1-amino­pyridinium iodide, followed by microwave-assisted nucleophilic aromatic substitution of iodo-cobaltocenium iodide. After anion exchange with potassium hexa­fluorido­phosphate, the title compound crystallizes as orange prisms in the space group *Pc*. This very stable pyridine nitrene adduct is the first example of a cobaltocenium derivative, formally containing a nitrene nitro­gen species.

## Abstract 2

The title compound was prepared in a microwave-assisted synthesis and is the first example of a cobaltocenium derivative formally containing a nitrene nitro­gen species.

## Graphical abstract

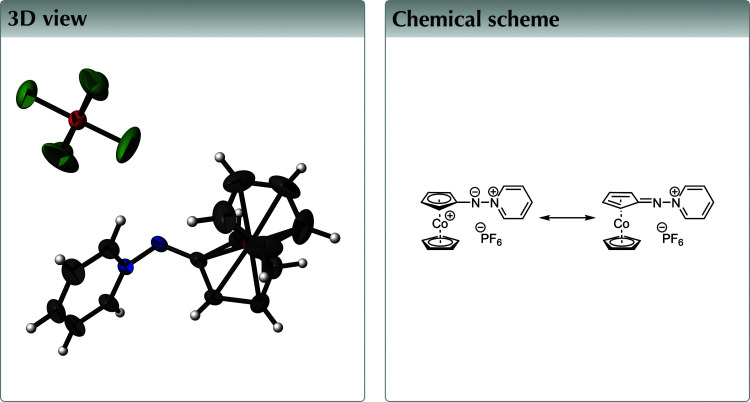

## Structure description

**Figure 1:**
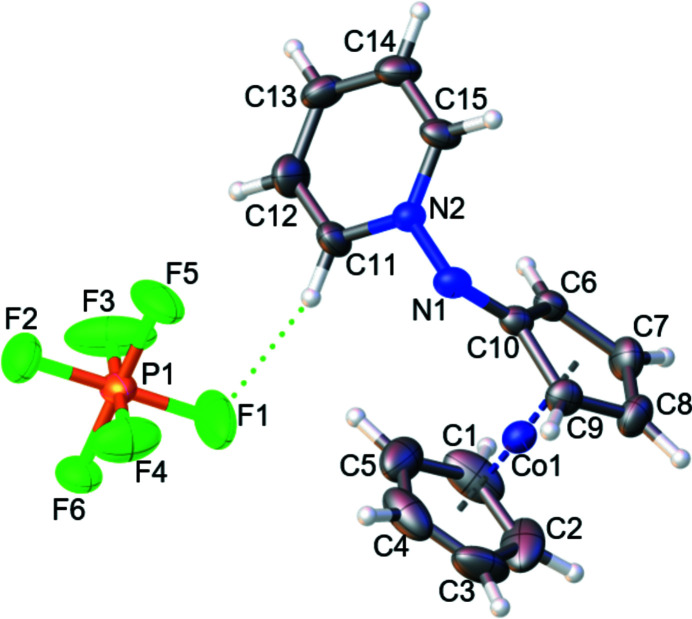
The molecular structure of the title compound, with displacement ellipsoids drawn at the 50% probability level for non-H atoms. Hydrogen bond H⋯F is represented by a green dashed line.

**Figure 2:**
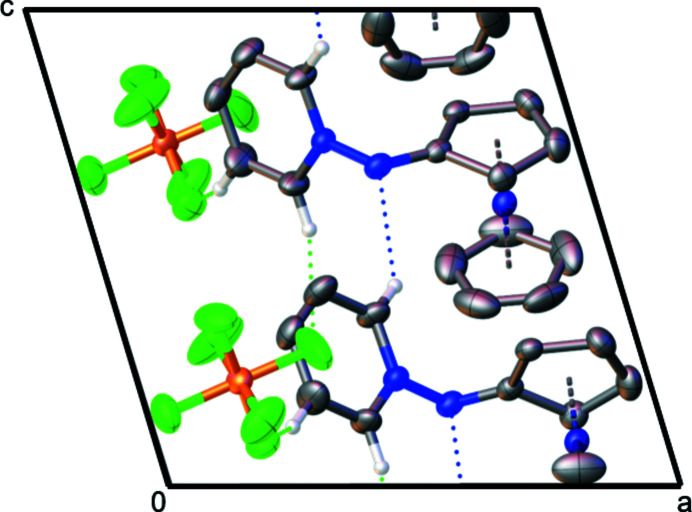
The arrangement of the mol­ecular units of the title compound in the unit cell, with displacement ellipsoids drawn at the 50% probability level for non-H atoms along the *b* axis. Hydrogen bonds are represented by dashed lines (H⋯N blue, H⋯F green). Hydrogen atoms not involved in hydrogen bonds are omitted for clarity. (Symmetry code: *x*, −*y*, *z* + 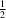 ).

**Figure 3:**
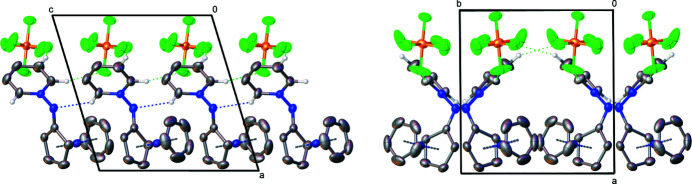
Formation of the hydrogen bonds of the title compound, with displacement ellipsoids drawn in at the 50% probability level for non-H atoms. Hydrogen bonds are represented by dashed lines (H⋯N blue, H⋯F green). Hydrogen atoms not involved in hydrogen bonds are omitted for clarity. Left view along the *b* axis, right view along the *c* axis.

The title compound (Fig. 1 ▸) is the first example of a cationic cobaltocenium nitrene species, stabilized by a bonded pyridine. It is highly polar, stable in various solvents up to high temperatures (approx. 200°C). The unsubstituted cyclo­penta­dienyl ring and the pyridine moiety are structurally as expected, displaying carbon–cobalt bond lengths for C1—C9 of 2.005 (7)–2.047 (5) Å and carbon–carbon C1—C15 lengths of 1.354 (9)–1.462 (7) Å, respectively. The substituted cyclo­penta­dienyl ring is slightly twisted out of plane [11,4(6)°] as the carbon–cobalt bond to C10 [2.227 (5) Å] is elongated. The bond lengths N1—N2 [1.421 (6) Å], N1—C10 [1.327 (7) Å] and bond angle C10—N1—N2 [110.4 (4)°], N1—N2—C11 [118.2 (4)°] are comparable to a penta­fluoro­phenyl (instead of cobaltocenium­yl) analogue (Poe *et al.*, 1992 ▸). Due to resonance, the N1—N2 and N1—C10 bond lengths are shortened compared to N—N [1.46 Å] and N—C [1.47 Å] standard single bonds. Weak hydrogen bonds (Table 1 ▸) are present between the anion and the pyridine substituent (Fig. 2 ▸) and inter­molecularly between the nitrene nitro­gen N1 and the pyridine H15, forming chains along the *c-*axis direction (Fig. 3 ▸).

## Synthesis and crystallization

In a microwave-assisted one-pot synthesis, first 9.44 g of 1-amino­pyridinium iodide (4.2 mmol, 1.5 equiv.) was deprotonated with 0.67 g of potassium *tert*-butoxide (5.9 mmol, 2.1 equiv.) in 100 ml of EtOH solution. Subsequently, after heating for 25 min (250 W, ramp 10 min, hold for 15 min, 100°C), 1.17 g of iodo-cobaltocenium iodide (Vanicek *et al.*, 2016 ▸) (2.8 mmol, 1 equiv.) were added and heating was continued for 40 min (250 W, ramp 10 min, hold for 30 min, 100°C). Workup: After cooling to room temperature, the mixture was transferred to a round-bottomed flask, 1.83 g of potassium hexa­fluorido­phosphate (9.9 mmol, 3.5 equiv.) were added and the mixture was stirred for 10 min. Neutral aluminium oxide (10 g) was added and the solvent was removed on a rotary evaporator. The product was purified, using a short neutral aluminium oxide column (*h* = 4 cm, *d* = 10 cm) with CH_3_CN as eluent. The solvent was removed on a rotary evaporator. The product was further dissolved in 200 ml CH_2_Cl_2_ and filtered. Toluene (20 ml) was added and the mixture was concentrated to 30 ml. Et_2_O (100 ml) was added and the product precipitated at −20°C over a period of 2 h. After filtration and washing with Et_2_O, 0.86 g of pure (cobaltoceniumyl­amido)­pyridinium hexa­fluorido­phosphate was obtained as an orange–red powder. Yield: 82% based on iodo­cobaltocenium iodide. M.p. 139–140 °C. HRMS (ESI+): *m*/*z* calc. 281.0484 (*M*+), found 281.0473 (*M*+). ^1^H NMR (400 MHz, CD_3_CN): δ 8.55 (*d x q*, *J* = 6.5, 1.3 Hz, 2H), 8.21 (*t x t*, *J* = 7.6, 1.3 Hz, 1H), 7.97-7.90 (*m*, 2H), 5.35 (*t*, *J* = 2.1 Hz, 2H), 5.26 (*s*, 5H), 4.63-4.56 (*m*, 2H). ^13^C NMR (75 MHz, CD_2_Cl_2_): δ 141.8, 139.0, 129.7, 105.1, 83.0, 77.6. Single crystals were obtained by vapor diffusion crystallization in acetone with Et_2_O at 4°C.

## Refinement

Crystal data, data collection and structure refinement details are summarized in Table 2 ▸.

## Supplementary Material

Crystal structure: contains datablock(s) global, I. DOI: 10.1107/S2414314621004600/bv4038sup1.cif

Structure factors: contains datablock(s) I. DOI: 10.1107/S2414314621004600/bv4038Isup2.hkl

CCDC reference: 2081212

Additional supporting information:  crystallographic information; 3D view; checkCIF report

## Figures and Tables

**Table 1:** Hydrogen-bond geometry (Å, °)

*D*—H⋯*A*	*D*—H	H⋯*A*	*D*⋯*A*	*D*—H⋯*A*
C11—H11⋯F1	0.95	2.35	3.273 (8)	164
C12—H12⋯F6^i^	0.95	2.46	3.293 (7)	146
C14—H14⋯F4^ii^	0.95	2.61	3.388 (7)	139
C15—H15⋯N1^iii^	0.95	2.44	3.156 (6)	132

Symmetry codes: (i) 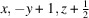 ; (ii) 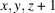 ; (iii) 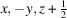 .

**Table 2:** Experimental details

Crystal data	
Chemical formula	[Co(C_5_H_5_)(C_10_H_9_N_2_)]PF_6_
*M*_r_	426.18
Crystal system, space group	Monoclinic, *P* *c*
Temperature (K)	183
*a*, *b*, *c* (Å)	9.9610 (8), 8.9243 (7), 9.7204 (7)
β (°)	106.943 (3)
*V* (Å^3^)	826.59 (11)
*Z*	2
Radiation type	Mo *K*α
μ (mm^−1^)	1.20
Crystal size (mm)	0.18 × 0.14 × 0.04

Data collection	
Diffractometer	Bruker D8 QUEST PHOTON 100
Absorption correction	Multi-scan (*SADABS*; Bruker, 2014 ▸)
*T*_min_, *T*_max_	0.817, 0.901
No. of measured, independent and observed [*I* > 2σ(*I*)] reflections	11210, 3329, 2999
*R*_int_	0.035
(sin θ/λ)_max_ (Å^−1^)	0.628

Refinement	
*R*[*F* ^2^ > 2σ(*F* ^2^)], *wR*(*F* ^2^), *S*	0.037, 0.093, 1.05
No. of reflections	3329
No. of parameters	227
No. of restraints	2
H-atom treatment	H-atom parameters constrained
Δρ_max_, Δρ_min_ (e Å^−3^)	1.01, −0.26
Absolute structure	Flack *x* determined using 1289 quotients [(*I* ^+^)−(*I* ^−^)]/[(*I* ^+^)+(*I* ^−^)] (Parsons *et al.*, 2013 ▸)
Absolute structure parameter	−0.007 (7)

Computer programs: *APEX3* and *SAINT* (Bruker, 2014 ▸), *SHELXT2014/4* (Sheldrick, 2015*a* ▸), *SHELXL2014/7* (Sheldrick, 2015*b* ▸), *OLEX2* (Dolomanov *et al.*, 2009 ▸) and *publCIF* (Westrip, 2010 ▸).

## full crystallographic data

### Crystal data

**Table:**

[Co(C_5_H_5_)(C_10_H_9_N_2_)]PF_6_	*F*(000) = 428
*M**_r_* = 426.18	*D*_x_ = 1.712 Mg m^−^^3^
Monoclinic, *P**c*	Mo *K*α radiation, λ = 0.71073 Å
*a* = 9.9610 (8) Å	Cell parameters from 5116 reflections
*b* = 8.9243 (7) Å	θ = 2.3–26.5°
*c* = 9.7204 (7) Å	µ = 1.20 mm^−^^1^
β = 106.943 (3)°	*T* = 183 K
*V* = 826.59 (11) Å^3^	Prism, orange
*Z* = 2	0.18 × 0.14 × 0.04 mm

### Data collection

**Table:**

Bruker D8 QUEST PHOTON 100 diffractometer	3329 independent reflections
Radiation source: Incoatec Microfocus	2999 reflections with *I* > 2σ(*I*)
Multi layered optics monochromator	*R*_int_ = 0.035
Detector resolution: 10.4 pixels mm^-1^	θ_max_ = 26.5°, θ_min_ = 2.1°
φ and ω scans	*h* = −12→12
Absorption correction: multi-scan (SADABS; Bruker, 2014)	*k* = −11→11
*T*_min_ = 0.817, *T*_max_ = 0.901	*l* = −11→12
11210 measured reflections	

### Refinement

**Table:**

Refinement on *F*^2^	Hydrogen site location: inferred from neighbouring sites
Least-squares matrix: full	H-atom parameters constrained
*R*[*F*^2^ > 2σ(*F*^2^)] = 0.037	*w* = 1/[σ^2^(*F*_o_^2^) + (0.0512*P*)^2^ + 0.4385*P*] where *P* = (*F*_o_^2^ + 2*F*_c_^2^)/3
*wR*(*F*^2^) = 0.093	(Δ/σ)_max_ < 0.001
*S* = 1.05	Δρ_max_ = 1.01 e Å^−^^3^
3329 reflections	Δρ_min_ = −0.26 e Å^−^^3^
227 parameters	Extinction correction: SHELXL-2014/7 (Sheldrick 2014), Fc^*^=kFc[1+0.001xFc^2^λ^3^/sin(2θ)]^-1/4^
2 restraints	Extinction coefficient: 0.015 (3)
Primary atom site location: structure-invariant direct methods	Absolute structure: Flack *x* determined using 1289 quotients [(*I*^+^)-(*I*^-^)]/[(*I*^+^)+(*I*^-^)] (Parsons *et al.*, 2013)
Secondary atom site location: difference Fourier map	Absolute structure parameter: −0.007 (7)

### Special details

**Table:**

Geometry. All esds (except the esd in the dihedral angle between two l.s. planes) are estimated using the full covariance matrix. The cell esds are taken into account individually in the estimation of esds in distances, angles and torsion angles; correlations between esds in cell parameters are only used when they are defined by crystal symmetry. An approximate (isotropic) treatment of cell esds is used for estimating esds involving l.s. planes.
Refinement. C-bound H atoms were placed in calculated positions and constrained to ride on their parent atoms with *U*_iso_(H) = 1.2*U*_eq_(C) and a C—H distance of 0.95 Å for aromatic H atoms. Refinement was performed using all reflections. The weighted *R*-factor (*wR*) and goodness of fit (S) are based on F^2^. *R*-factor (gt) are based on F. The threshold expression of F^2^ > 2.0 sigma(F^2^) is used only for calculating *R*-factor (gt).

### Fractional atomic coordinates and isotropic or equivalent isotropic displacement parameters (Å^2^)

**Table:**

	*x*	*y*	*z*	*U*_iso_*/*U*_eq_	
Co1	0.82275 (6)	0.26234 (6)	0.59021 (6)	0.0254 (2)	
N1	0.5977 (5)	0.0343 (4)	0.6695 (4)	0.0274 (9)	
N2	0.5060 (4)	0.1237 (4)	0.7231 (4)	0.0252 (9)	
C1	0.8101 (10)	0.4810 (7)	0.5333 (7)	0.063 (2)	
H1	0.8231	0.5613	0.6000	0.075*	
C2	0.9142 (8)	0.4111 (9)	0.4857 (9)	0.068 (2)	
H2	1.0108	0.4372	0.5104	0.081*	
C3	0.8468 (10)	0.2939 (9)	0.3934 (8)	0.060 (2)	
H3	0.8918	0.2237	0.3481	0.072*	
C4	0.7089 (10)	0.2978 (9)	0.3801 (8)	0.059 (2)	
H4	0.6405	0.2326	0.3215	0.070*	
C5	0.6824 (8)	0.4094 (9)	0.4636 (8)	0.058 (2)	
H5	0.5930	0.4348	0.4732	0.070*	
C6	0.7849 (6)	0.2298 (6)	0.7833 (6)	0.0287 (12)	
H6	0.7348	0.3021	0.8204	0.034*	
C7	0.9298 (7)	0.2339 (7)	0.7978 (7)	0.0396 (15)	
H7	0.9954	0.3032	0.8544	0.048*	
C8	0.9606 (6)	0.1167 (6)	0.7131 (7)	0.0397 (13)	
H8	1.0505	0.0934	0.7034	0.048*	
C9	0.8336 (6)	0.0404 (6)	0.6455 (6)	0.0319 (12)	
H9	0.8223	−0.0353	0.5744	0.038*	
C10	0.7242 (6)	0.0961 (5)	0.7018 (5)	0.0256 (10)	
C11	0.4222 (6)	0.2215 (6)	0.6334 (6)	0.0377 (13)	
H11	0.4244	0.2300	0.5367	0.045*	
C12	0.3324 (7)	0.3100 (8)	0.6834 (8)	0.0501 (16)	
H12	0.2748	0.3822	0.6218	0.060*	
C13	0.3266 (7)	0.2939 (7)	0.8201 (7)	0.0422 (14)	
H13	0.2659	0.3552	0.8554	0.051*	
C14	0.4102 (7)	0.1871 (7)	0.9076 (7)	0.0420 (14)	
H14	0.4051	0.1722	1.0028	0.050*	
C15	0.4995 (6)	0.1035 (6)	0.8571 (6)	0.0331 (13)	
H15	0.5574	0.0305	0.9172	0.040*	
P1	0.19726 (16)	0.24876 (16)	0.21938 (17)	0.0344 (4)	
F1	0.3620 (5)	0.2643 (7)	0.2863 (6)	0.105 (2)	
F2	0.0334 (4)	0.2311 (4)	0.1507 (5)	0.0554 (11)	
F3	0.1747 (7)	0.3921 (5)	0.3041 (6)	0.095 (2)	
F4	0.2192 (5)	0.1044 (5)	0.1347 (5)	0.0743 (14)	
F5	0.1885 (5)	0.1453 (5)	0.3502 (4)	0.0668 (14)	
F6	0.2058 (4)	0.3523 (4)	0.0880 (4)	0.0452 (9)	

### Atomic displacement parameters (Å^2^)

**Table:**

	*U*^11^	*U*^22^	*U*^33^	*U*^12^	*U*^13^	*U*^23^
Co1	0.0257 (3)	0.0225 (3)	0.0292 (4)	−0.0010 (4)	0.0101 (2)	−0.0013 (4)
N1	0.034 (2)	0.025 (2)	0.027 (2)	0.0000 (17)	0.0146 (19)	−0.0016 (16)
N2	0.026 (2)	0.026 (2)	0.025 (2)	0.0014 (17)	0.0092 (17)	0.0012 (16)
C1	0.114 (7)	0.023 (3)	0.044 (4)	−0.004 (3)	0.014 (4)	0.003 (2)
C2	0.041 (4)	0.074 (5)	0.084 (6)	−0.018 (4)	0.013 (4)	0.041 (5)
C3	0.096 (7)	0.055 (4)	0.039 (4)	0.027 (5)	0.035 (4)	0.007 (3)
C4	0.081 (6)	0.045 (4)	0.038 (4)	−0.023 (4)	−0.002 (4)	0.011 (3)
C5	0.049 (4)	0.059 (4)	0.070 (5)	0.019 (3)	0.023 (4)	0.038 (4)
C6	0.034 (3)	0.031 (3)	0.022 (3)	0.004 (2)	0.009 (2)	0.001 (2)
C7	0.031 (3)	0.044 (4)	0.039 (4)	−0.004 (3)	0.004 (3)	−0.002 (3)
C8	0.027 (3)	0.039 (3)	0.052 (4)	0.007 (2)	0.011 (3)	0.006 (3)
C9	0.035 (3)	0.025 (2)	0.037 (3)	0.004 (2)	0.013 (3)	−0.0003 (19)
C10	0.033 (3)	0.022 (2)	0.024 (2)	0.006 (2)	0.012 (2)	0.0046 (18)
C11	0.039 (3)	0.047 (3)	0.027 (3)	0.012 (3)	0.009 (2)	0.012 (2)
C12	0.045 (4)	0.058 (4)	0.049 (4)	0.026 (3)	0.015 (3)	0.021 (3)
C13	0.040 (3)	0.045 (3)	0.049 (4)	0.009 (3)	0.025 (3)	0.005 (3)
C14	0.052 (4)	0.044 (3)	0.039 (4)	0.009 (3)	0.028 (3)	0.010 (3)
C15	0.046 (3)	0.031 (3)	0.026 (3)	0.008 (2)	0.017 (3)	0.009 (2)
P1	0.0312 (8)	0.0438 (10)	0.0295 (8)	−0.0074 (6)	0.0108 (6)	0.0027 (6)
F1	0.042 (3)	0.183 (7)	0.074 (4)	−0.042 (3)	−0.009 (2)	0.055 (3)
F2	0.034 (2)	0.064 (2)	0.064 (3)	−0.0091 (17)	0.0072 (18)	0.0112 (19)
F3	0.162 (6)	0.064 (3)	0.096 (4)	−0.056 (3)	0.096 (4)	−0.046 (3)
F4	0.104 (4)	0.049 (2)	0.094 (4)	0.011 (2)	0.067 (3)	0.001 (2)
F5	0.072 (3)	0.089 (3)	0.037 (2)	−0.029 (3)	0.012 (2)	0.019 (2)
F6	0.050 (2)	0.049 (2)	0.0397 (19)	−0.0074 (16)	0.0184 (17)	0.0105 (16)

### Geometric parameters (Å, º)

**Table:**

Co1—C7	2.005 (7)	C6—C7	1.408 (9)
Co1—C8	2.012 (6)	C6—C10	1.462 (7)
Co1—C3	2.016 (7)	C6—H6	0.9500
Co1—C1	2.022 (6)	C7—C8	1.419 (9)
Co1—C6	2.040 (6)	C7—H7	0.9500
Co1—C2	2.041 (7)	C8—C9	1.418 (8)
Co1—C9	2.047 (5)	C8—H8	0.9500
Co1—C5	2.047 (6)	C9—C10	1.442 (7)
Co1—C4	2.051 (7)	C9—H9	0.9500
Co1—C10	2.227 (5)	C11—C12	1.383 (9)
N1—C10	1.327 (7)	C11—H11	0.9500
N1—N2	1.421 (6)	C12—C13	1.355 (9)
N2—C15	1.335 (6)	C12—H12	0.9500
N2—C11	1.340 (7)	C13—C14	1.383 (9)
C1—C2	1.399 (11)	C13—H13	0.9500
C1—C5	1.408 (11)	C14—C15	1.358 (8)
C1—H1	0.9500	C14—H14	0.9500
C2—C3	1.414 (12)	C15—H15	0.9500
C2—H2	0.9500	P1—F3	1.572 (5)
C3—C4	1.342 (12)	P1—F4	1.578 (4)
C3—H3	0.9500	P1—F2	1.581 (4)
C4—C5	1.358 (12)	P1—F1	1.585 (5)
C4—H4	0.9500	P1—F5	1.595 (4)
C5—H5	0.9500	P1—F6	1.599 (4)
			
C7—Co1—C8	41.4 (3)	C3—C4—H4	125.3
C7—Co1—C3	142.9 (3)	C5—C4—H4	125.3
C8—Co1—C3	113.8 (3)	Co1—C4—H4	126.5
C7—Co1—C1	111.7 (3)	C4—C5—C1	108.3 (7)
C8—Co1—C1	139.8 (3)	C4—C5—Co1	70.8 (4)
C3—Co1—C1	67.7 (3)	C1—C5—Co1	68.8 (4)
C7—Co1—C6	40.8 (3)	C4—C5—H5	125.8
C8—Co1—C6	68.8 (2)	C1—C5—H5	125.8
C3—Co1—C6	176.3 (4)	Co1—C5—H5	126.1
C1—Co1—C6	112.1 (3)	C7—C6—C10	109.0 (5)
C7—Co1—C2	113.6 (3)	C7—C6—Co1	68.3 (4)
C8—Co1—C2	112.9 (3)	C10—C6—Co1	77.1 (3)
C3—Co1—C2	40.8 (4)	C7—C6—H6	125.5
C1—Co1—C2	40.3 (3)	C10—C6—H6	125.5
C6—Co1—C2	141.2 (3)	Co1—C6—H6	120.8
C7—Co1—C9	69.0 (2)	C6—C7—C8	108.1 (5)
C8—Co1—C9	40.9 (2)	C6—C7—Co1	71.0 (3)
C3—Co1—C9	111.9 (3)	C8—C7—Co1	69.6 (4)
C1—Co1—C9	179.3 (3)	C6—C7—H7	125.9
C6—Co1—C9	68.3 (2)	C8—C7—H7	125.9
C2—Co1—C9	139.8 (3)	Co1—C7—H7	125.1
C7—Co1—C5	139.1 (3)	C9—C8—C7	107.9 (5)
C8—Co1—C5	179.5 (3)	C9—C8—Co1	70.9 (3)
C3—Co1—C5	65.7 (3)	C7—C8—Co1	69.0 (3)
C1—Co1—C5	40.5 (3)	C9—C8—H8	126.1
C6—Co1—C5	111.6 (3)	C7—C8—H8	126.1
C2—Co1—C5	67.0 (3)	Co1—C8—H8	125.6
C9—Co1—C5	138.8 (3)	C8—C9—C10	109.3 (5)
C7—Co1—C4	177.8 (4)	C8—C9—Co1	68.2 (3)
C8—Co1—C4	140.8 (3)	C10—C9—Co1	77.2 (3)
C3—Co1—C4	38.5 (4)	C8—C9—H9	125.4
C1—Co1—C4	66.8 (3)	C10—C9—H9	125.4
C6—Co1—C4	137.8 (3)	Co1—C9—H9	120.8
C2—Co1—C4	66.5 (3)	N1—C10—C9	122.5 (4)
C9—Co1—C4	112.5 (3)	N1—C10—C6	133.0 (5)
C5—Co1—C4	38.7 (3)	C9—C10—C6	104.3 (5)
C7—Co1—C10	66.8 (2)	N1—C10—Co1	133.2 (3)
C8—Co1—C10	66.5 (2)	C9—C10—Co1	63.6 (3)
C3—Co1—C10	138.3 (3)	C6—C10—Co1	63.2 (3)
C1—Co1—C10	140.8 (3)	N2—C11—C12	119.2 (5)
C6—Co1—C10	39.8 (2)	N2—C11—H11	120.4
C2—Co1—C10	178.8 (3)	C12—C11—H11	120.4
C9—Co1—C10	39.14 (19)	C13—C12—C11	120.0 (6)
C5—Co1—C10	113.5 (3)	C13—C12—H12	120.0
C4—Co1—C10	113.2 (3)	C11—C12—H12	120.0
C10—N1—N2	110.4 (4)	C12—C13—C14	119.1 (6)
C15—N2—C11	121.6 (5)	C12—C13—H13	120.4
C15—N2—N1	120.1 (4)	C14—C13—H13	120.4
C11—N2—N1	118.2 (4)	C15—C14—C13	119.8 (6)
C2—C1—C5	106.9 (6)	C15—C14—H14	120.1
C2—C1—Co1	70.6 (4)	C13—C14—H14	120.1
C5—C1—Co1	70.7 (4)	N2—C15—C14	120.1 (5)
C2—C1—H1	126.5	N2—C15—H15	119.9
C5—C1—H1	126.5	C14—C15—H15	119.9
Co1—C1—H1	123.8	F3—P1—F4	179.7 (3)
C1—C2—C3	106.2 (7)	F3—P1—F2	90.8 (3)
C1—C2—Co1	69.1 (4)	F4—P1—F2	88.9 (3)
C3—C2—Co1	68.7 (4)	F3—P1—F1	90.1 (4)
C1—C2—H2	126.9	F4—P1—F1	90.1 (3)
C3—C2—H2	126.9	F2—P1—F1	179.0 (3)
Co1—C2—H2	126.8	F3—P1—F5	90.2 (3)
C4—C3—C2	109.0 (7)	F4—P1—F5	89.5 (3)
C4—C3—Co1	72.1 (4)	F2—P1—F5	89.4 (2)
C2—C3—Co1	70.5 (4)	F1—P1—F5	90.8 (2)
C4—C3—H3	125.5	F3—P1—F6	89.8 (2)
C2—C3—H3	125.5	F4—P1—F6	90.4 (2)
Co1—C3—H3	123.4	F2—P1—F6	90.5 (2)
C3—C4—C5	109.5 (7)	F1—P1—F6	89.3 (2)
C3—C4—Co1	69.3 (4)	F5—P1—F6	179.9 (3)
C5—C4—Co1	70.5 (4)		
			
C10—N1—N2—C15	−87.4 (5)	Co1—C8—C9—C10	−66.9 (4)
C10—N1—N2—C11	96.1 (5)	C7—C8—C9—Co1	59.3 (4)
C5—C1—C2—C3	−2.8 (7)	N2—N1—C10—C9	−171.9 (4)
Co1—C1—C2—C3	58.9 (5)	N2—N1—C10—C6	2.6 (7)
C5—C1—C2—Co1	−61.7 (4)	N2—N1—C10—Co1	−89.0 (5)
C1—C2—C3—C4	3.1 (8)	C8—C9—C10—N1	−172.7 (4)
Co1—C2—C3—C4	62.3 (5)	Co1—C9—C10—N1	126.1 (4)
C1—C2—C3—Co1	−59.2 (5)	C8—C9—C10—C6	11.4 (5)
C2—C3—C4—C5	−2.1 (9)	Co1—C9—C10—C6	−49.7 (3)
Co1—C3—C4—C5	59.2 (5)	C8—C9—C10—Co1	61.2 (4)
C2—C3—C4—Co1	−61.3 (5)	C7—C6—C10—N1	173.5 (5)
C3—C4—C5—C1	0.3 (8)	Co1—C6—C10—N1	−125.2 (5)
Co1—C4—C5—C1	58.8 (5)	C7—C6—C10—C9	−11.3 (6)
C3—C4—C5—Co1	−58.5 (5)	Co1—C6—C10—C9	50.0 (3)
C2—C1—C5—C4	1.7 (7)	C7—C6—C10—Co1	−61.3 (4)
Co1—C1—C5—C4	−60.0 (5)	C15—N2—C11—C12	3.8 (9)
C2—C1—C5—Co1	61.7 (5)	N1—N2—C11—C12	−179.8 (6)
C10—C6—C7—C8	7.0 (7)	N2—C11—C12—C13	−2.1 (11)
Co1—C6—C7—C8	−59.9 (4)	C11—C12—C13—C14	−0.7 (11)
C10—C6—C7—Co1	66.9 (4)	C12—C13—C14—C15	2.0 (11)
C6—C7—C8—C9	0.3 (7)	C11—N2—C15—C14	−2.6 (9)
Co1—C7—C8—C9	−60.5 (4)	N1—N2—C15—C14	−178.9 (5)
C6—C7—C8—Co1	60.8 (4)	C13—C14—C15—N2	−0.4 (10)
C7—C8—C9—C10	−7.6 (6)		

### Hydrogen-bond geometry (Å, º)

**Table:**

*D*—H···*A*	*D*—H	H···*A*	*D*···*A*	*D*—H···*A*
C11—H11···F1	0.95	2.35	3.273 (8)	164
C12—H12···F6^i^	0.95	2.46	3.293 (7)	146
C14—H14···F4^ii^	0.95	2.61	3.388 (7)	139
C15—H15···N1^iii^	0.95	2.44	3.156 (6)	132

Symmetry codes: (i) *x*, −*y*+1, *z*+1/2; (ii) *x*, *y*, *z*+1; (iii) *x*, −*y*, *z*+1/2.
